# Supplementary material for: Identification and Purification of Novel Low-Molecular-Weight Lupine Allergens as Components for Personalized Diagnostics
Source: Nutrients. 2021 Jan 28;13(2):409. doi: 10.3390/nu13020409 (PMC7911308; doi:10.3390/nu13020409)
Supplement: Supplementary file 1 [file nutrients-13-00409-s001.pdf]

Supplementary materials
Figures

Figure S 1 A-E

**A. Nucleotide and protein sequence from database entry FG090100**	
Accession number	**FG090100** (EST database), for sequence see below
g ggg ata tca ctc act ctc tat ctc ttt ctc tca tag att ttc att tca aag cta<br>G I S L T L Y L F L S - I F I S K L<br>agt ttg aga aaa atg **tcg tgg caa acc tac gtt gat gag cac ctt ttg tgc gat**<br>S L R K M **S W Q T Y V D E H L L C D**<br>**atc gaa ggt aat cag ctc act tct gca gcc att atc ggc caa gac ggt agc gtt**<br>**I E G N Q L T S A A I I G Q D G S V**<br>**tgg gct cag agc tct tct ttc cct cag ttc aag cct gag gaa ata act gct atc**<br>**W A Q S S S F P Q F K P E E I T A I**<br>**gtg aat gac ttt gct gag cct gga tca ctt gct cca act gga tta tat ctc ggt**<br>**V N D F A E P G S L A P T G L Y L G**<br>**ggc acg aaa tat atg gtg atc caa ggt gag cca gga gct gtc att cga ggg aaa**<br>**G T K Y M V I Q G E P G A V I R G K**<br>**aag ggt cct ggt ggg gtt act gtt aag aag acc aat caa gct ttg atc att ggt**<br>**K G P G G V T V K K T N Q A L I I G**<br>**att tat gat gaa cca atg act cca ggc caa tgc aac gtg gta gtt gaa agg ctt**<br>**I Y D E P M T P G Q C N V V V E R L**<br>**ggt gat tat ctc att gat acc ggt ctt** taa tcc tct tgg taa ttc ttg tta tcg<br>**G D Y L I D T G L** - S S W - F L L S<br>ttc tta ttt cat tgg ctt tta taa gag gct ttt aca tca act tgc cat gag aat<br>F L F H W L L - E A F T S T C H E N<br>tct tga ttg caa tgt aat aat att atg gct at<br>S - L Q C N N I M A	

**B. Nucleotide and protein sequence used for expression**	
cat atg **tcg tgg caa acc tac gtt gat gag cac ctt ttg tgc gat**<br>H M **S W Q T Y V D E H L L C D**<br>**atc gaa ggt aat cag ctc act tct gca gcc att atc ggc caa gac ggt agc gtt**<br>**I E G N Q L T S A A I I G Q D G S V**<br>**tgg gct cag agc tct tct ttc cct cag ttc aag cct gag gaa ata act gct atc**<br>**W A Q S S S F P Q F K P E E I T A I**<br>**gtg aat gac ttt gct gag cct gga tca ctt gct cca act gga tta tat ctc ggt**<br>**V N D F A E P G S L A P T G L Y L G**<br>**ggc acg aaa tat atg gtg atc caa ggt gag cca gga gct gtc att cga ggg aaa**<br>**G T K Y M V I Q G E P G A V I R G K**<br>**aag ggt cct ggt ggg gtt act gtt aag aag acc aat caa gct ttg atc att ggt**<br>**K G P G G V T V K K T N Q A L I I G**<br>**att tat gat gaa cca atg act cca ggc caa tgc aac gtg gta gtt gaa agg ctt**<br>**I Y D E P M T P G Q C N V V V E R L**<br>**ggt gat tat ctc att gat acc ggt ctt** ctc gag<br>**G D Y L I D T G L** L E	
Cleavage sites:	NdeI (cat atg); marked in blue<br>XhoI (ctc gag); marked in blue
Expression system:	Expression system: *E. coli* BL21(DE3),<br>Vector: pET-23b

C. Final protein sequence (after expression)	
Incl. His-Tag (black)	SWQTYVDEHLLCDIEGNQLTSAAIIGQDGSVWAQSSSFPQFKPEEITAIVNDFAEPGSLAPTGLYLGGTKYMVIQGEPGAVIRGKKGPGGVTVKKTNQALIIGIYDEPMTPGQCNVVVERLGDYLIDTGL**LEHHHHHH**

D. Molecular weight of the expressed protein	
Deduced from sequence	13.849 kDa (without His-Tag)
SDS-PAGE (reduced)	15 kDa (with His-Tag)

E. N-terminal sequencing	
sequence coverage (bold):	**SWQTYVDEHL**LCDIEGNQLTSAAIIGQDGSVWAQSSSFPQFKPEEITAIVNDFAEPGSLAPTGLYLGGTKYMVIQGEPGAVIRGKKGPGGVTVKKTNQALIIGIYDEPMTPGQCNVVVERLGDYLIDTGLLEHHHHHH

Figure S 1 A-E. Identification of the sequence information on *L. albus* (A) available from the EST data base, (B) sequence used for recombinant expression of the authentic protein and (C-E) preparation for mass spectrometry analysis

Figure S 2 A-E

A. Mass spectrometry of the recombinant FG090100 *L. albus* protein (profilin) – Full MS

Full MS (deconvoluted)

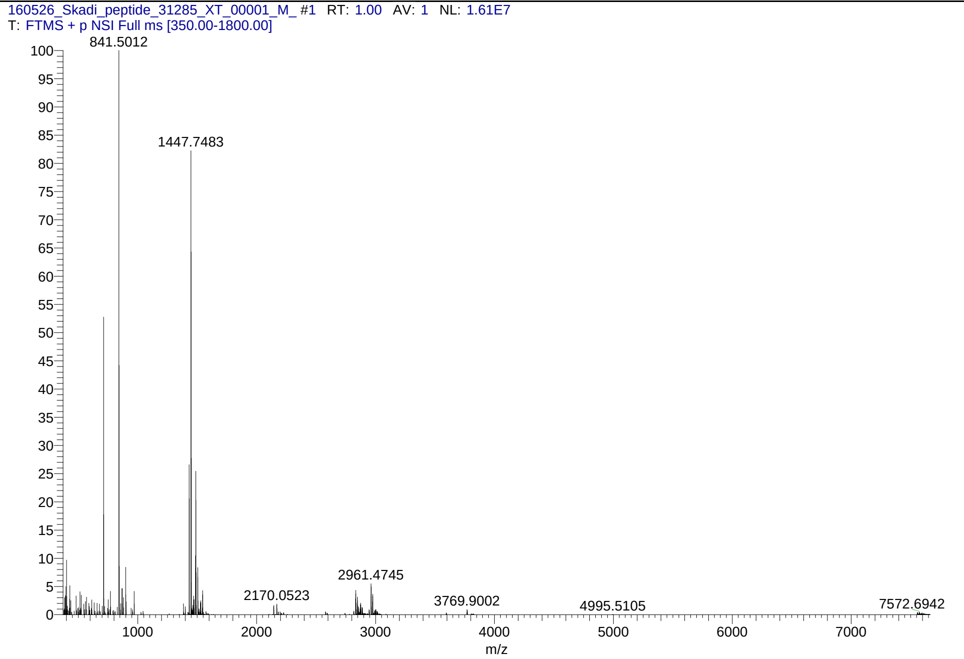

Full-MS masses:

**Mass** determined	**mass** thero.	**peptide**	**Pos.**	**Mod.**	**MC**
713.4067	713.4072	GPGGVTVK	87-94	-	0
841.5012	841.5021	KGPGGVTVK	86-94	-	1
1431.7538	1431.7544	YMVIQGEPGAVIR	71-83	-	0
1447.7483	1447.7493	YMVIQGEPGAVIR	71-83	MSO:72	0
2143.0335	2143.0347	LGDYLIDTGLLEHHHHHH	121-138	-	0
3832.3772	2832.3786	TNQALIIGIYDEPMTPGQCNVV VER	96-120	1xCys_CAM, 1xMSO	0
2960.4702	2960.4735	KTNQALIIGIYDEPMTPGQCNV VVER	95-120	1xCys_CAM, 1xMSO	1

sequence coverage (bold **~47.8 %**):	SWQTYVDEHLLCDIEGNQLTSAAIIGQDGSVWAQSSSFPQFKPEEITAIVNDFAEPGSL APTGLYLGGTK**YMVIQGEPGAVIR**GK**KGPGGVTVKKTNQALIIGIYDEPMTPGQCNVVV ERLGDYLIDTGLLEHHHHHH**

B. Mass spectrometry of natural *L. albus* protein (profilin) – Full MS

1D-PAGE

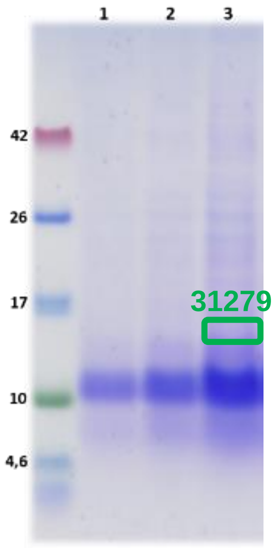

1D-PAGE - L. albus extract

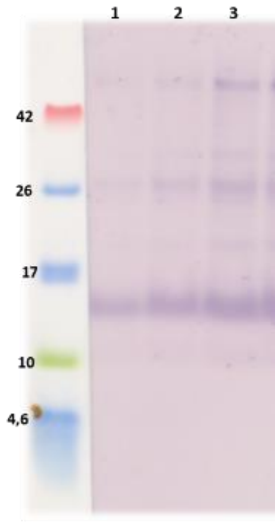

1D-Immunoblot of *L. albus* extract developed with serum P3

Full MS (**31279**) (deconvoluted)

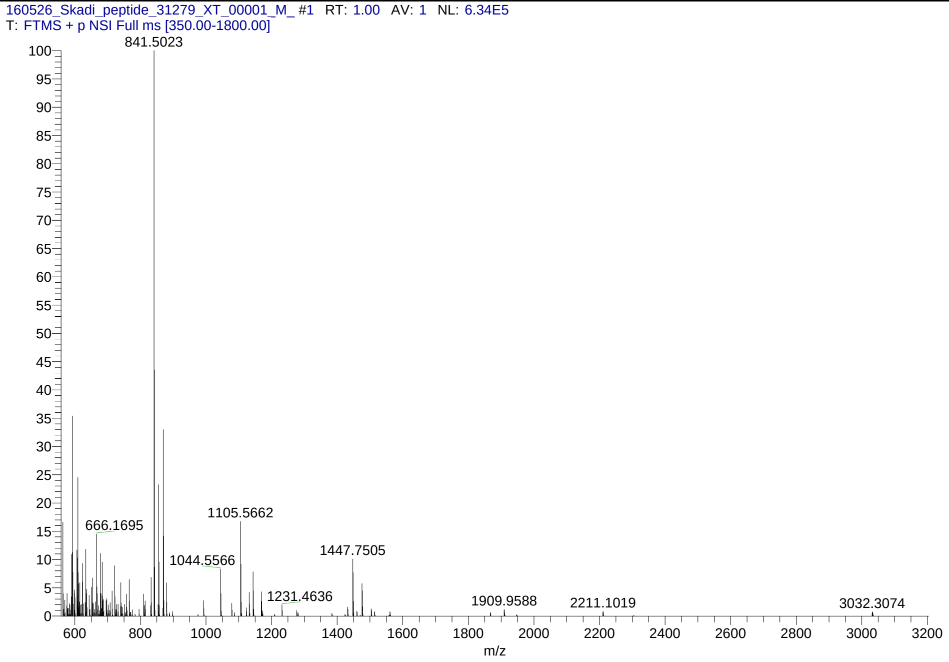

Full-MS masses:

**Mass** determined	**mass** thero.	**peptide**	**Pos.**	**Mod.**	**MC**
1447.7505	1447.7493	YMVIQGEPGAVIR	71-83	MSO:72	0

C. Mass spectrometry of natural *L. albus* protein (profilin) – MS/MS

MS/MS of fragment	**YMVIQGEPGAVIR**
MS/MS spectrum **31279** of m/z 724.88 $[M+H]^{2+}$	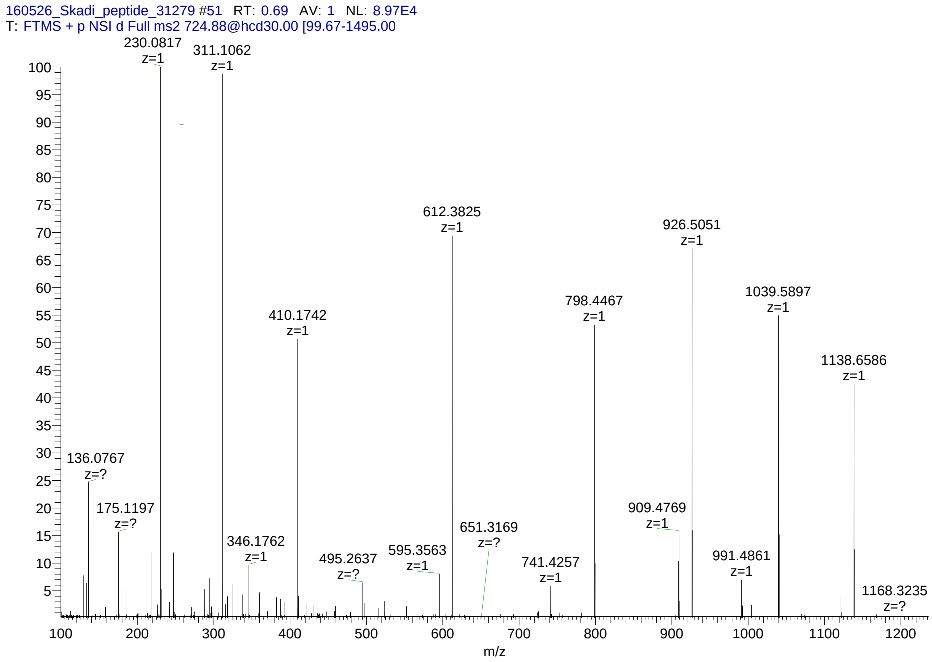

MS/MS masses m/z 724.88 $[M+H]^{2+}$:

**Mass y-ion** determined	**Mass y-ion** thero.	**Amino acid**
---	---	**Y**
---	1285.6933	**M (oxidation)**
1138.6586	1138.6579	**V**
1039.5897	1039.5895	**I**
926.5051	926.5054	**Q**
798.4467	798.4468	**G**
741.4257	741.4254	**E**
612.3825	612.3828	**P**
515.3312	515.3300	**G**
458.3088	458.3085	**A**
387.2711	387.2714	**V**
288.2032	288.2030	**I**
175.1197	175.1190	**R**

D. Mass spectrometry of natural *L. albus* protein (profilin) – Full MS

2D-PAGE

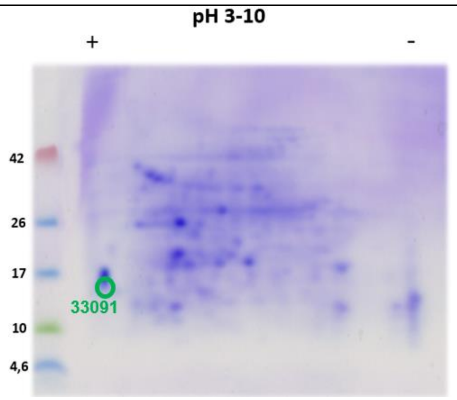

2D-PAGE - *L. albus* extract

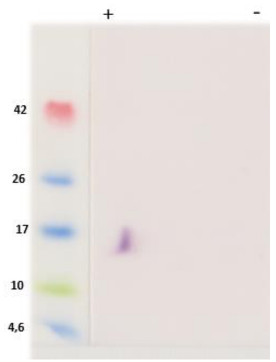

2D-Immunoblot of *L. albus* extract developed with serum P4

Full MS (33091) (deconvoluted)

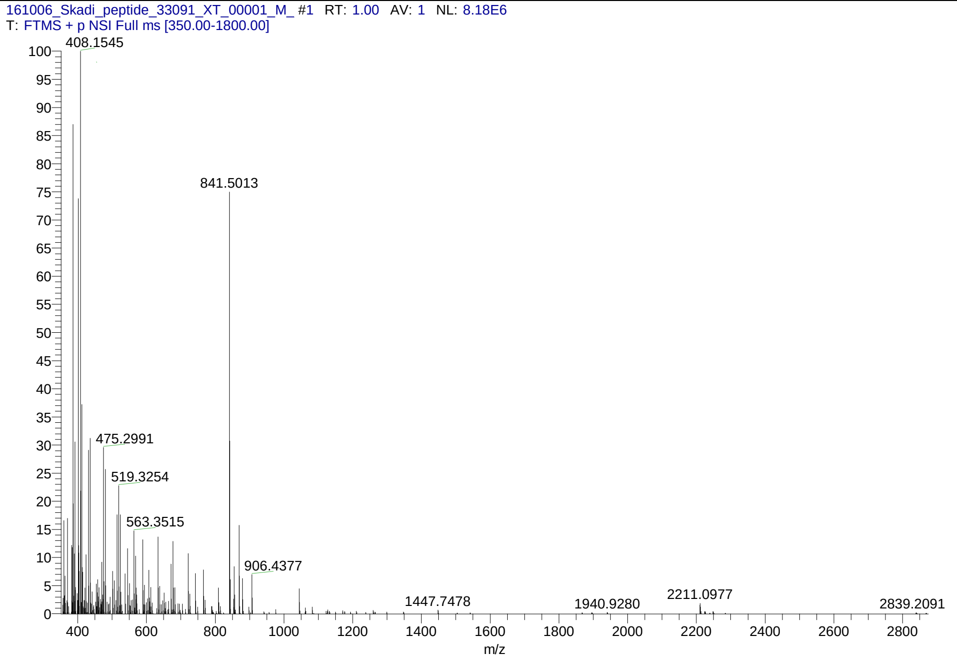

Full-MS masses:

Mass determined	mass thero.	peptide	Pos.	Mod.	MC
1447.7478	1447.7493	YMVIQGEPGAVIR	71-83	MSO:72	0

sequence coverage (bold):

SWQTYVDEHLLCDIEGNQLTSAAIIGQDGSVWAQSSSFPQFKPEEITAIVNDFAEPGSLAPTGLYLGGTK**YMVIQGEPGAVIR**GKKGPGGVTVKKTNQALIIGIYDEPMTPGQCNVVVERLGDYLIDTGL

E. Mass spectrometry of natural *L. albus* protein (profilin) – MS/MS

MS/MS of fragment	**YMVIQGEPGAVIR**
MS/MS spectrum **33091** of m/z 724.88 $[M+H]^{2+}$	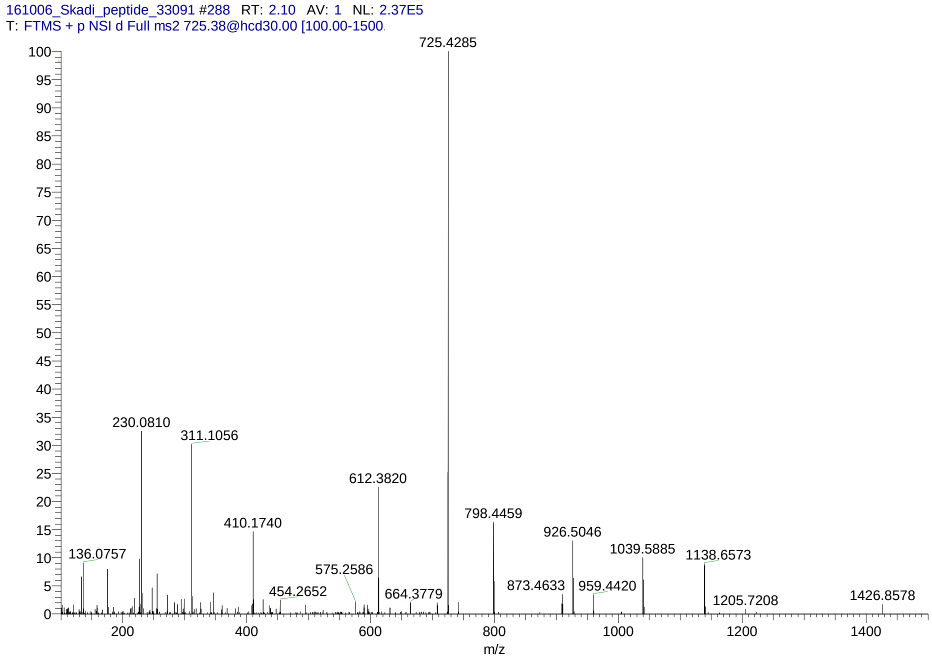

MS/MS masses m/z 724.88 $[M+H]^{2+}$:

**Mass y-ion** determined	**Mass y-ion** thero.	**Amino acid**
---	---	**Y**
---	1285.6933	**M (oxidation)**
1138.6573	1138.6579	**V**
1039.5885	1039.5895	**I**
926.5046	926.5054	**Q**
798.4459	798.4468	**G**
741.4247	741.4254	**E**
612.3820	612.3828	**P**
515.3301	515.3300	**G**
-	458.3085	**A**
387.2707	387.2714	**V**
288.2028	288.2030	**I**
175.1189	175.1190	**R**

As submitted to the WHO/IUIS allergen nomenclature sub-committee, 2016

Figure S 2 A-E. Synopsis of the mass spectrometry analyses of *L. albus* recombinant profilin and the *L. albus* profilin purified from the natural source.

Figure S 3 A-D

A. Database protein sequence (XP_019446786.1)

PREDICTED: non-specific lipid-transfer protein 1-like [Lupinus angustifolius]

NCBI Reference Sequence: XP_019446786.1

Identical Proteins FASTA Graphics

Go to:

```
LOCUS       XP_019446786             116 aa            linear   PLN 16-DEC-2016
DEFINITION  PREDICTED: non-specific lipid-transfer protein 1-like [Lupinus
            angustifolius].
ACCESSION   XP_019446786
VERSION     XP_019446786.1
DBLINK      BioProject: PRJNA356456
DBSOURCE    REFSEQ: accession XM_019591241.1
KEYWORDS    RefSeq.
SOURCE      Lupinus angustifolius (narrow-leaved blue lupine)
  ORGANISM  Lupinus angustifolius
            Eukaryota; Viridiplantae; Streptophyta; Embryophyta; Tracheophyta;
            Spermatophyta; Magnoliophyta; eudicotyledons; Gunneridae;
            Pentapetalae; rosids; fabids; Fabales; Fabaceae; Papilionoideae;
            Genisteae; Lupinus.
COMMENT     MODEL REFSEQ:  This record is predicted by automated computational
            analysis. This record is derived from a genomic sequence
            (NC_032009.1) annotated using gene prediction method: Gnomon.
            Also see:
                Documentation of NCBI's Annotation Process

            ##Genome-Annotation-Data-START##
            Annotation Provider         :: NCBI
            Annotation Status           :: Full annotation
            Annotation Version          :: Lupinus angustifolius Annotation
                                           Release 100
            Annotation Pipeline         :: NCBI eukaryotic genome annotation
                                           pipeline
            Annotation Software Version :: 7.2
            Annotation Method           :: Best-placed RefSeq; Gnomon
            Features Annotated          :: Gene; mRNA; CDS; ncRNA
            ##Genome-Annotation-Data-END##
            COMPLETENESS: full length.
FEATURES             Location/Qualifiers
     source          1..116
                     /organism="Lupinus angustifolius"
                     /cultivar="Tanjil"
                     /db_xref="taxon:3871"
                     /chromosome="LG01"
                     /tissue_type="whole plant"
                     /dev_stage="seedling"
                     /country="Australia: Western Australia"
     Protein         1..116
                     /product="non-specific lipid-transfer protein 1-like"
                     /calculated_mol_wt=11589
     CDS             1..116
                     /gene="LOC109350101"
                     /coded_by="XM_019591241.1:78..428"
                     /db_xref="GeneID:109350101"
ORIGIN
        1 magivklaca vlicmvvvsa pltkaitcgq vtanlaqcln ylrsggavpa pccngiknil
       61 nlakttpdrr tacnclkaaa antpglnpsn agslpgkcgv nipykistst ncasik
//
```

>XP_019446786.1 PREDICTED: non-specific lipid-transfer protein 1-like [Lupinus angustifolius]
MAGIVKLACAVLICMVVVSAPLTKA**ITCGQVTANLAQCLNYLRSGGAVPAPCCNGIKNILNLAKTTPDRR**
TACNCLKAAAANTPGLNPSNAGSLPGKCGVNIPYKISTSTNCASIK
Signal peptide, mature sequence

B. Molecular weight of the mature protein

1D-Page	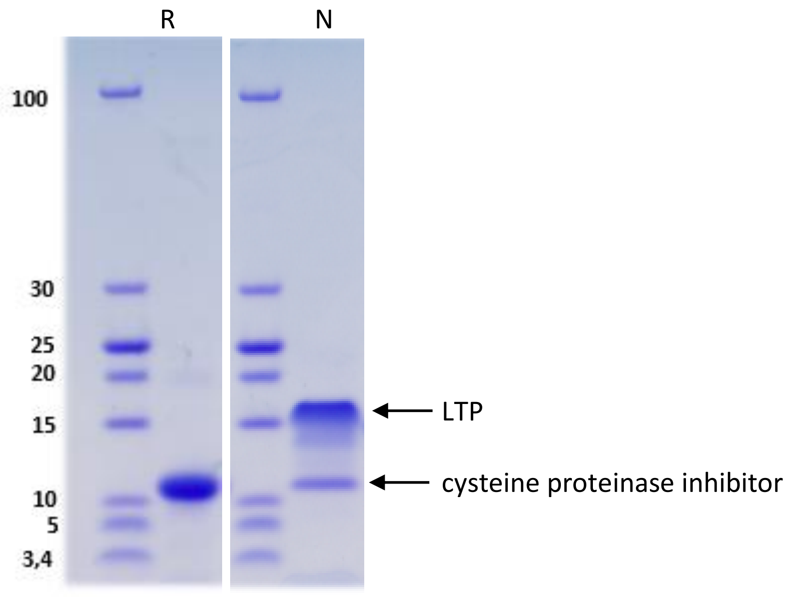 SDS-PAGE of *L. angustifolius* LTP under reducing (R) and non-reducing (N) conditions
Deduced from sequence	9.23 kDa (monoisotopic mass)
SDS-PAGE (reduced)	~11.00 kDa
SDS-PAGE (non-reduced)	~16.00 kDa

C. N-terminal sequencing

sequence coverage (bold):13.2%	**ITCGQVTANLAQ**CLNYLRSGGAVPAPCCNGIKNILNLAKTTPDRRTACNCLKAAAANTP GLNPSNAGSLPGKCGVNIPYKISTSTNCASIK

D. Mass spectrometry of natural *L. angustifolius* protein (LTP) – Full MS

1D-Page

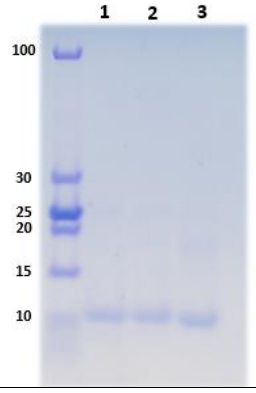

Full MS (deconvoluted)

Spectra from sample 1 (lane 1)

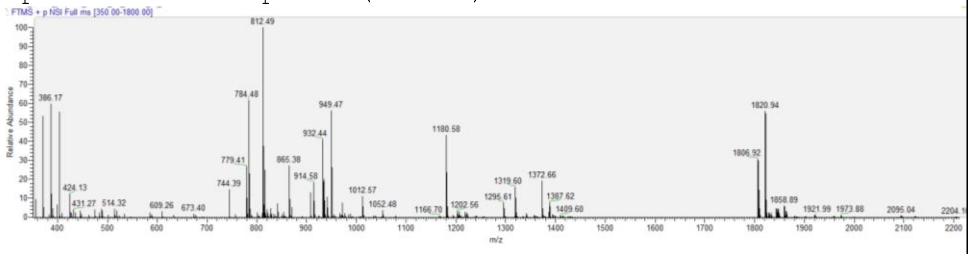

Spectra from sample 2 (lane 2)

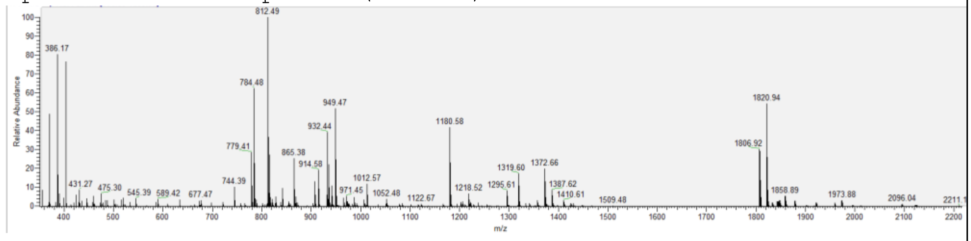

Spectra from sample 3 (lane 3)

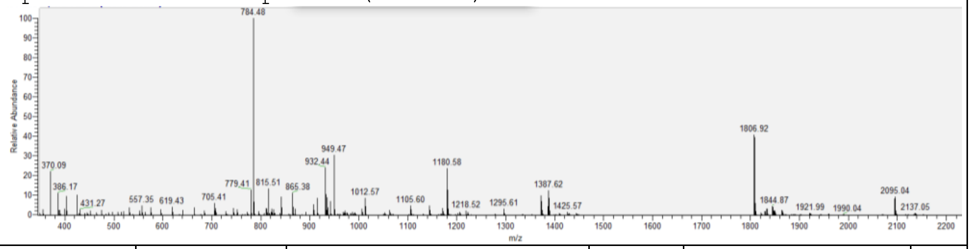

Full-MS masses:

Mass determined	mass thero.	peptide	Pos.	Mod.	MC
744.3872	744.3878	TTPDRR	39-45	-	1
784.4799	784.4807	NILNLAK	33-38	-	-
865.3780	865.3786	TACNCLK	46-52	carbamidomethyl	-
949.4685	949.4691	CGVNIPYK	73-80	carbamidomethyl	-
1180.5752	1180.5758	ISTSTNCASIK	80-91	carbamidomethyl	-
1386.6378	1386.6384	SGGAVPAPCCNGIK	19-32	carbamidomethyl	-
1806.9214	1806.9224	AAAANTPGLNPSNAGSLPGK	53-72	-	-
2095.0389	2095.0350	ITCGQVTANLAQCLNYLR	1-18	carbamidomethyl	-

sequence coverage (bold 100%):

ITCGQVTANLAQCLNYLRSGGAVPAPCCNGIKNILNLAKTTPDRRTACNCLKAAAANTP GLNPSNAGSLPGKCGVNIPYKISTSTNCASIK

As submitted to the WHO/IUIS allergen nomenclature sub-committee, 25/09/2019**.**

Figure S 3 A-D. *Lupinus angustifolius* lipid transfer protein (LTP): (A) database protein sequence, (B) molecular weight determination of the mature protein (under reducing and non-reducing conditions), (C) N-terminal sequencing, and (D) mass spectrometry.
